# Add-on Pyridostigmine Enhances CD4^+^ T-Cell Recovery in HIV-1-Infected Immunological Non-Responders: A Proof-of-Concept Study

**DOI:** 10.3389/fimmu.2017.01301

**Published:** 2017-10-18

**Authors:** Sergio I. Valdés-Ferrer, José C. Crispín, Pablo F. Belaunzarán-Zamudio, Carlos A. Rodríguez-Osorio, Bernardo Cacho-Díaz, Jorge Alcocer-Varela, Carlos Cantú-Brito, Juan Sierra-Madero

**Affiliations:** ^1^Departamento de Neurología, Instituto Nacional de Ciencias Médicas y Nutrición Salvador Zubirán, Mexico City, Mexico; ^2^Departamento de Infectología, Instituto Nacional de Ciencias Médicas y Nutrición Salvador Zubirán, Mexico City, Mexico; ^3^Center for Biomedical Science, Feinstein Institute for Medical Research, Manhasset, NY, United States; ^4^Departamento de Inmunología y Reumatología, Instituto Nacional de Ciencias Médicas y Nutrición Salvador Zubirán, Mexico City, Mexico; ^5^Departamento de Medicina Crítica, Instituto Nacional de Ciencias Médicas y Nutrición Salvador Zubirán, Mexico City, Mexico; ^6^Department of Molecular Biology, Massachusetts General Hospital, Boston, MA, United States; ^7^Departamento de Neurología, Instituto Nacional de Cancerología, Mexico City, Mexico

**Keywords:** HIV, pyridostigmine, CD4^+^ T-cell, immune reconstitution, cholinergic anti-inflammatory pathway

## Abstract

**Background:**

In human immunodeficiency virus (HIV)-infection, persistent T-cell activation leads to rapid turnover and increased cell death, leading to immune exhaustion and increased susceptibility to opportunistic infections. Stimulation of the vagus nerve increases acetylcholine (ACh) release and modulates inflammation in chronic inflammatory conditions, a neural mechanism known as the *cholinergic anti-inflammatory pathway* (CAP). Pyridostigmine (PDG), an ACh-esterase inhibitor, increases the half-life of endogenous ACh, therefore mimicking the CAP. We have previously observed that PDG reduces *ex vivo* activation and proliferation of T-cells obtained from people living with HIV.

**Methods:**

We conducted a 16-week proof-of-concept open trial using PDG as add-on therapy in seven HIV-infected patients with discordant immune response receiving combined antiretroviral therapy, to determine whether PDG would promote an increase in total CD4^+^ T-cells. The trial was approved by the Institutional Research and Ethics Board and registered in ClinicalTrials.gov (NCT00518154).

**Results:**

Seven patients were enrolled after signing informed consent forms. We observed that addition of PDG induced a significant increase in total CD4^+^ T-cells (baseline = 153.1 ± 43.1 vs. week-12 = 211.9 ± 61.1 cells/µL; *p* = 0.02). *Post hoc* analysis showed that in response to PDG, four patients (57%) significantly increased CD4^+^ T-cell counts (responders = 257.8 ± 26.6 vs. non-responders = 150.6 ± 18.0 cells/µL; *p* = 0.002), and the effect persisted for at least 1 year after discontinuation of PDG.

**Conclusion:**

Our data indicate that in patients with HIV, add-on PDG results in a significant and persistent increase in circulating CD4^+^ T-cells.

## Introduction

Effective suppression of viral replication with the use of antiretroviral therapy (cART) leads to recovery of circulating CD4^+^ T-cells (1, 2). Around 20% of patients starting cART with advanced disease show an insufficient increase of CD4^+^ T-cells, even after adequate suppression of viral replication, reflecting an incomplete immune reconstitution (3, 4). Those patients, called *immunological non-responders* (INRs), have increased long-term susceptibility to opportunistic infections, as well as accelerated disease progression and mortality (5). Various mechanisms have been proposed to underlie incomplete immune reconstitution, including older age, higher baseline HIV-RNA, low baseline CD4^+^ counts (≤50 cells/μL), poor adherence to therapy, and enhanced CD4^+^ cell activation, all leading to cell dysfunction and apoptosis (6).

The cholinergic anti-inflammatory pathway (CAP) is a neural mechanism that modulates inflammation through the release of acetylcholine (ACh), resulting in decreased synthesis of inflammatory cytokines such as TNF-α and IL-1 (7). Pyridostigmine (PDG), a peripherally acting ACh-esterase inhibitor used for the treatment of patients with myasthenia gravis, increases ACh bioavailability. We recently showed that in HIV-infected patients, PDG reduces T-cell overactivation and proliferation, reduces production of IFN-γ, and increases production of IL-10 (8).

We therefore hypothesized that in HIV-infected patients with complete HIV-RNA suppression, but incomplete CD4^+^ T-cell reconstitution, adding PDG to cART would promote the recovery of CD4^+^ T-cell counts by reducing T-cell overactivation.

## Methods

### Study Design

The study was investigator-generated and planned as a prospective, interventional, open label, proof-of-concept trial, to determine whether the addition of PDG to cART improved the immune status of HIV-1-infected subjects with optimal virological control but inadequate immune reconstitution. Eligible patients received PDG bromide [Mestinon^®^; 3-(dimethylaminocarbonyloxy)-1-methylpyridinium bromide; CAS number: 101-26-8] 30 mg, TID, PO as add-on to their usual cART for 16 weeks. At each visit, participants were asked about compliance, tolerability, and adverse effects. Then, patients received a refill of PDG to cover the following 4-week period plus a 7-day supply. Delivery of PDG was done at the outpatient Infectious Diseases Clinic.

### Ethical Considerations

The trial was approved by the Institutional Review Board and by the Ethics Committee (registered as Ref-1663 and Ref-1873) of the Instituto Nacional de Ciencias Médicas y Nutrición Salvador Zubirán. The study was also registered in a public registry (ClinicalTrials.gov identifier: NCT00518154). All participants signed a written informed consent, keeping a copy themselves. Valeant Pharmaceuticals donated the PDG tablets but had no role on study design, analysis, or publication of results.

### Patients

**Figure 1:**
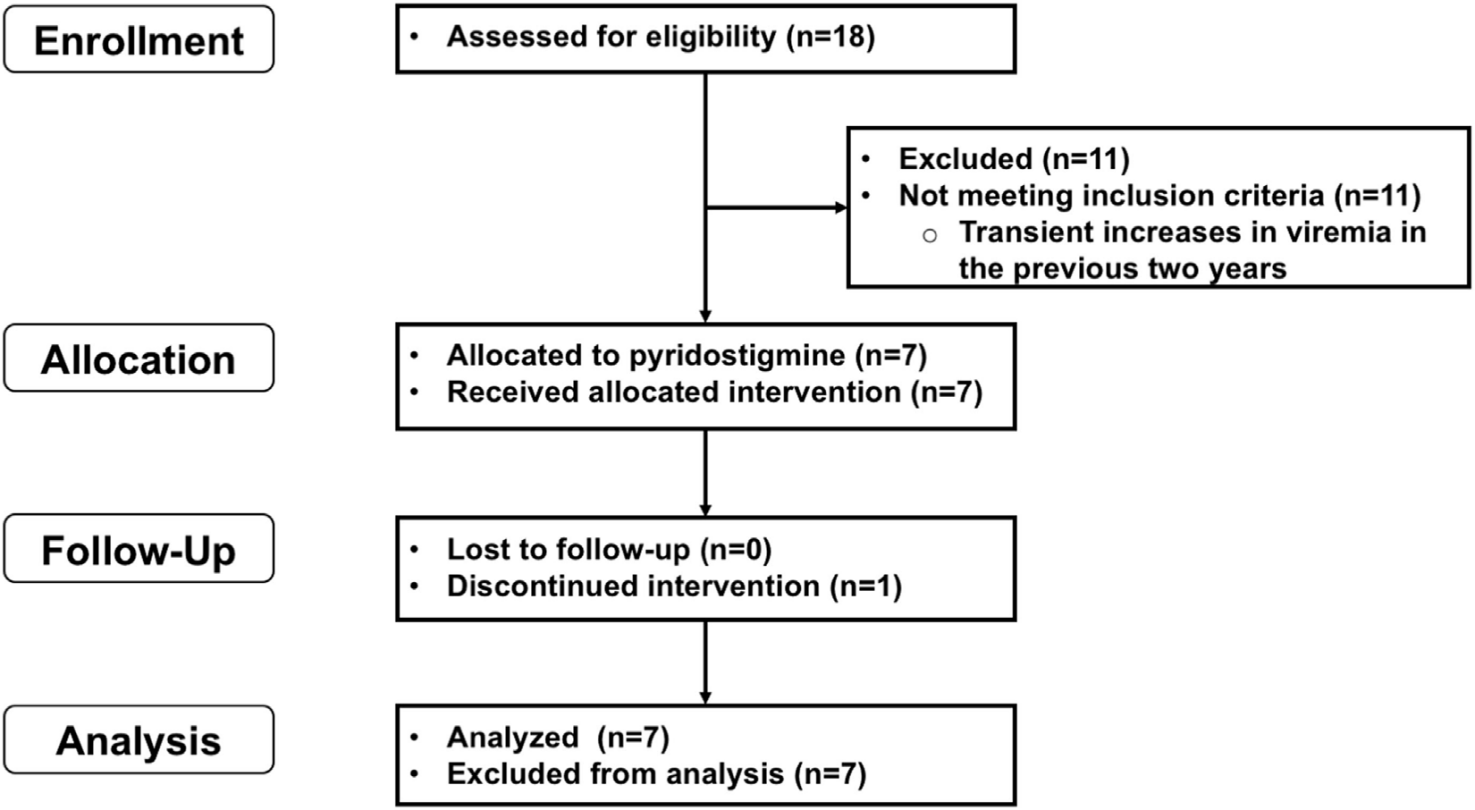
CONSORT flowchart of patient enrollment, follow-up, and analysis.

We invited adult patients (≥18 years), carriers of HIV-1 infection being followed at the HIV/AIDS Clinic of our Institute. Patients had to be taking effective cART, as evidenced by suppressed viremia under a constant therapeutic regime for at least 2 years, but with incomplete immunological response. We defined incomplete immunological response as failure to increase at least 50 CD4^+^ cells/μL/year during the first 2 years of virological control; failure to reach a CD4^+^ cell count of 200/µL; or a combination of both. A flowchart including participant selection, recruitment, and analysis is presented as Figure 1.

A complete blood count (CBC) as well as CD4^+^ and CD8^+^ T-cell quantification were carried at pre-established time points: 8, 12, and 16 weeks of PDG; 4 weeks after the last dose of PDG (*washout* period); and, 1 year after the last intake of PDG. Patients were blinded to their response to PDG during the study period. Exclusion criteria included the following: (a) concomitant active or neoplastic disease; (b) history of new AIDS-defining illness during cART; (c) pregnancy or breast feeding; (d) patients who had been subjects of an investigational agent, chemotherapy, or radiotherapy within the previous 28 days; (e) active tuberculosis or other opportunistic infections; (f) that participants were unable to follow or comply with the protocol interventions; and (g) that participants were receiving immunosuppressive or immunomodulatory drugs, including corticosteroids.

### Measured Outcomes

The primary outcome was the number of circulating CD4^+^ T-cells at different time points in comparison with the baseline level. Secondary readouts included (a) ratio of baseline-to-peak CD4^+^ cell counts and (b) changes in total T-cell numbers. CD4^+^ and CD8^+^ T-cells were quantified by flow cytometry as described previously (8). Analysis of samples was carried at a core facility, where technicians were unaware of sample source.

### Statistical Analysis

Being a proof-of-concept study, and aiming to include all eligible subjects, we had no sample size or power estimation. Exploratory data analysis was made using graphic methods. We describe baseline demographic, clinical, and laboratory characteristics of participants using means as measure of central tendency and its corresponding SDs. We did an unplanned analysis to search for factors associated with adequate immune response using the same definition of adequate response used for inclusion criteria. We carried out an intention-to-treat analysis to include the single participant who stopped experimental medication early. We present statistics of the whole group and of stratified patients according to their immune response to PDG administration. We compared the differences in CD4^+^ T-cell concentrations at baseline, and at weeks 8, 12, and 16 using paired *t-tests* and summarize these changes using graphical methods. We compared mean measured CD4^+^ T-cells at each time point between patients with ANOVA. Statistical analysis was done on Stata (release 12) or GraphPad Prism (version 5).

## Results

### Patients

Eighteen patients fulfilling the inclusion criteria were screened and invited to participate. Eleven patients had had transient elevations in viremia during the 2 years before recruitment and were excluded. The remaining seven patients were enrolled after signing informed consent. All were male and infected through unprotected sex with males (MSM). All were free of co-infections, including hepatotropic viruses, tuberculosis, or opportunistic pathogens. Baseline characteristics of the patients can be found in Table 1. In brief, patients had been diagnosed as HIV-positive for a mean of 76.6 months (6.3 years) and, on average had delayed the start of antiretroviral therapy for almost 3 years. Except for persistently low CD4^+^ cells (153.2 ± 43.1 cells/µL), participants had CBCs within the normal range (Table 1).

**Table 1:** Baseline characteristics of participants according to response status.

	Patients		

	All (*n* = 7)	Responders (*n* = 4)	Non-responders (*n* = 3)

	*N* (SD)	*N* (SD)	*N* (SD)
**Baseline demographic data**			
Age at inclusion and start of PDG (years)	48.1 (11.1)	42.6 (9.20)	55.6 (10.1)
Age at diagnosis (years)	41.7 (12.3)	37.3 (11.4)	47.6 (12.8)
Diagnosis to cART span (months)	35.0 (53.2)	26.6 (41.4)	46.2 (74.8)
HAART to PDG span (months)	41.6 (20.3)	35.9 (10.9)	49.1 (30.1)
Diagnosis to start of PDG (months)	76.6 (68.0)	62.5 (41.4)	95.4 (101.8)
Circulating CD4^+^ T cells at diagnosis (cells/µL)	73.0 (66.2)	64.3 (40.3)	84.7 (101.8)
Circulating CD4^+^ T cells at start of cART (cells/µL)	77.4 (43.5)	74.3 (17.6)	81.7 (71.8)
**CBC at initiation of pyridostigmine**			
Hemoglobin (g/dL)	15.0 (0.7)	15.5 (0.9)	14.7 (0.4)
Hematocrit (%)	42.6 (1.9)	44.4 (2)	41.7 (1.3)
WBC (×10^3^/µL)	5.6 (1.8)	4.4 (0.3)	6.4 (2.0)
Granulocytes (×10^3^/µL)	3.8 (1.8)	4.5 (2.2)	2.9 (0.3)
Monocytes (×10^3^/µL)	0.4 (0.1)	0.4 (0.1)	0.4 (0.1)
Lymphocytes (×10^3^/µL)	1.2 (0.6)	1.4 (0.8)	1.1 (0.2)
Circulating CD4^+^ T cells (cells/µL)	153.2 (43.1)	170.3 (43.3)	130.3 (37.0)

*Data expressed as follows: mean (±SD) or median (p25–p75)*.

*cART, combined antiretroviral therapy; PDG, pyridostigmine*.

### Antiretroviral Therapy and Virological Control

Four subjects were taking boosted protease inhibitor (PI)-based regimens (three lopinavir and one atazanavir), and three were receiving non-nucleoside transcriptase inhibitor (NNRTI)-based regimens (efavirenz). Five subjects were receiving their first cART regimen (three NNRTI-based and one PI-based). Throughout the study, all HIV load determinations were below detection level (Roche 1.5 ultra-sensitive).

### Add-on PDG

Six patients reported full adherence to PDG. One patient (on the Responder group, as described below) took PDG for the first 12 weeks, and then withdrew consent for reasons unrelated to the study, but accepted to have CBC and CD4^+^ determinations taken from the charts up to the predetermined 1-year posttrial time points. Adverse reactions to PDG were monitored daily during the first week, then weekly during the first month and every 4 weeks thereafter. No cardiovascular, respiratory, gastrointestinal, genitourinary, or other reactions were identified. Monitoring of the central nervous system functions did not reveal any symptom of cholinergic side effects. Compliance relied on self-report.

### CD4^+^ Response to PDG

**Figure 2:**
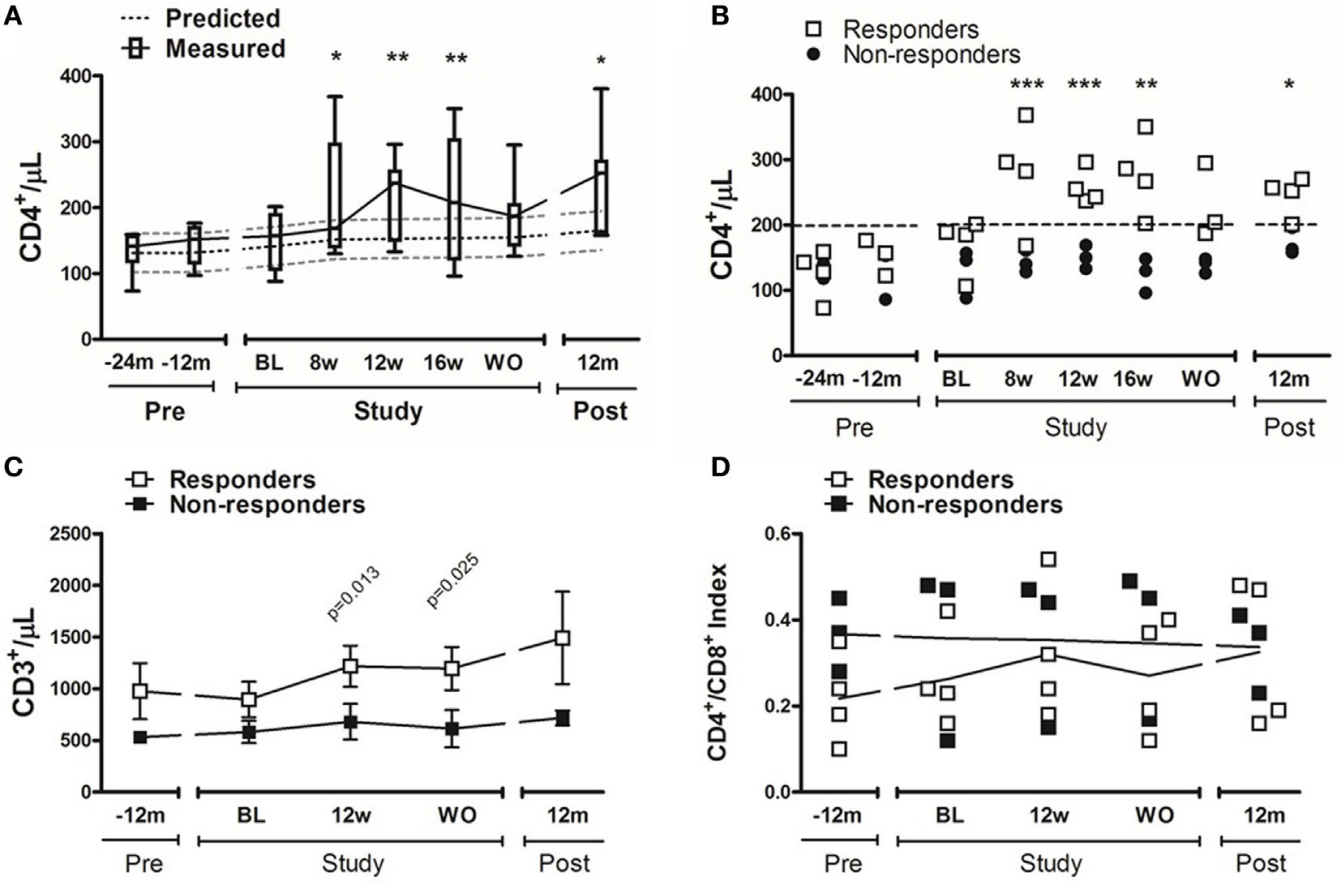
CD4^+^ cell increase before, during, and after pyridostigmine (PDG). Total CD4^+^ cell counts were determined by flow cytometry as CD3^+^, CD4^+^, and CD8^neg^ cells. **(A)** The solid line shows the dynamic increase of total CD4^+^ T-cells [median (p25–p75)] during PDG administration; for comparison, the broken lines depict predicted increase in circulating CD4^+^ cells of 35 cells/mL/year (4). **(B)** A subgroup of patients showed a significant, rapid, and persistent elevation in CD4^+^ cells, and this increase persisted after washout, and 1-year after PDG (each symbol represents and individual value). **(C)** Total T-cells were determined by flow cytometry as CD3^+^ cells (mean ± SD). **(D)** The ratio of CD4^+^ and CD8^+^ cells was determined by gating from total (CD3^+^) T-cells (each symbol represents and individual value). *n* = 7 for panel **(A)**; *n* = 3–4 subjects/group for panels **(B–D)**. *p ≤ 0.05; **p ≤ 0.01 vs. baseline.

We observed a significant increase in CD4^+^ T-cell counts in response to the addition of PDG (baseline = 153.2 ± 43.1 vs. week-12 = 211.9 ± 61.1 cells/μL; *p* = 0.02), that persisted for as long as PDG was taken. After discontinuing PDG, circulating CD4^+^ T-cell counts decreased to similar levels to those observed before inclusion. One year after the discontinuation of PDG, CD4^+^ T-cell counts were significantly elevated in comparison with baseline (baseline = 153.2 ± 43.1 vs. 1-year = 213.9 ± 45.9 cells/µL; *p* = 0.02) (Figure 2A).

We then performed a *post hoc* analysis, separating participants dichotomically according to CD4^+^ T-cell response: for that, we defined patients whose CD4^+^ T-cells reached 200 cells/μL as *responders*, and those who failed to achieve 200 cells/μL as *non-responders*. Responders separated from the non-responders early after they started taking PDG (Figure 2B). Moreover, among responders, the CD4^+^ T-cell counts remained significantly increased for the duration of intervention, returned to baseline levels after discontinuation of PDG. We observed a trend for higher baseline levels of total T-cells (CD3^+^) among responders than non-responders (895.5 ± 345.0 vs. 581.0 ± 186.8 cells/µL, respectively; *p* = 0.11); by week-12, responders had significantly higher total T-cell counts than non-responders (1,217.0 ± 396.7 vs. 681.0 ± 297.2 cells/µL, respectively; *p* = 0.05) (Figure 2C). The CD4^+^/CD8^+^ index remained stable during the study period for all subjects, suggesting that the increase in CD4^+^ cells was due to a global increase in T-cells rather than to an effect on CD4^+^ cells (Figure 2D).

While no individual characteristic predicted response, non-responders were older, had been diagnosed at an older age, had been untreated for HIV infection for twice as long after being diagnosed, and had lower mean CD4^+^ T-cells at baseline, suggesting that the lack of response might be due to age-related decreased thymic output (Table 1).

## Discussion

In this proof-of-concept paper, we present data suggesting that add-on therapy with the ACh-esterase inhibitor PDG improves the number of circulating CD4^+^ T-cells in HIV-infected, immunological non-responder patients (INRs).

In HIV infection, initiation of cART leads to reduced viremia, increased CD4^+^ T-cell counts, normalization of the CD4^+^/CD8^+^ ratio, and reduction in morbidity and mortality (3). Most patients using cART respond by effectively shutting down viremia and increasing CD4^+^ T-cell counts. Around 20% of patients starting cART with advanced disease show an insufficient increase of CD4^+^ cells even after adequate suppression of viral replication (9).

A CD4^+^ count below 200 cells/μL implies severe immunosuppression and precludes the discontinuation of primary prophylaxis against opportunistic infections (e.g., *Pneumocystis* pneumonia or *Toxoplasma gondii* encephalitis). The clinical consequences of being an INR are dire, because the severe CD4^+^ lymphopenia increases dramatically the risk of developing opportunistic infections and malignant disease (10, 11). Further, low CD4^+^ T-cell counts are associated with faster disease progression and mortality (5, 12, 13).

The mechanisms underlying CD4^+^ T-cell lymphopenia in patients infected with HIV are incompletely understood. Nonetheless, persistent immune activation, observed even in patients with suppressed viral replication, is considered a critical factor for CD4^+^ T-cell depletion (14). A number of pharmacological strategies, including hydroxychloroquine, statins, cyclosporine A, mycophenolic acid, and rapamycin, have unsuccessfully tried to improve the cellular response in HIV-infected patients (14–16). In a previous report, we showed that PDG is able to modulate T-cell activation—as reflected by cytokine production and cell proliferation—in patients with HIV infection (8). Based on those findings, we hypothesized that T-cell modulation through enhanced Ach availability could improve CD4^+^ T-cell counts in patients with HIV infection that had failed to accomplish adequate immune reconstitution.

In this study, more than half of the patients treated with PDG experienced an increase in CD4^+^ T-cell numbers of sufficient magnitude to allow them to cross the clinically relevant threshold of 200 cells/μL. Moreover, although the effect was greatest during exposure to the drug, it showed to be long lasting and a year after the discontinuation of the experimental therapy, the responders maintained CD4^+^ T-cell counts higher than 200 cells/μL.

The nervous system senses cytokines and other inflammatory signals and responds, through an anti-inflammatory reflex, *via* the vagus nerve. The efferent branch of this system is known as the CAP. Accordingly, electrical stimulation of the vagus nerve, or administration of cholinergic agonists, inhibit the inflammatory response and lower the mortality of experimental endotoxemia and other cytokine-mediated inflammation by inducing or facilitating the CAP (17). Likewise, activation of the CAP during systemic inflammation downregulates the production and release of inflammatory cytokines (18).

T-cells are affected by the CAP and accordingly, they express all the critical components for ACh signaling, including nicotinic and muscarinic receptors, as well as the enzymatic machinery needed for ACh synthesis (19–21). ACh can activate or suppress T-cells, depending on the kinetics of its stimulation. Brief stimulation of nicotinic receptors on T-cells results in Ca^2+^ signaling and cell activation, while sustained stimulation results in downregulation of T-cell activation (19, 22).

Pyridostigmine, a reversible ACh-esterase inhibitor, has been in use since the mid-1950s for symptomatic treatment of myasthenia gravis (23, 24) and as prophylactic agent against biological warfare involving neurotoxic agents (25, 26). By inhibiting ACh esterase, PDG increases the half-life of endogenous ACh. Because of its hydrophilic nature, its penetrance to the central nervous system is limited and thus it is considered the standard of care for myasthenia gravis and for civilians and soldiers at risk of exposure to nerve agents (e.g., sarin) (27, 28). The data presented here demonstrate that in principle, enhancement of ACh availability may have a clinically relevant effect in increasing CD4^+^ T-cell counts in a subset of INR patients with HIV infection.

As a pilot study, our work has many limitations, including the lack of a control group, a small sample size consisting only of male participants, and the use of only one dose of PDG. The choice of the PDG dose (90 mg/day) was based on previous reports finding less than 0.1% of serious side effects among 41,650 healthy soldiers taking PDG as prophylaxis against biological warfare (25). It is important to note, however, that as a proof-of-concept study, the present work was not designed to evaluate different doses. The optimal dose of PDG will have to be determined in future studies. Regarding gender imbalance, we invited all participants fulfilling our inclusion criteria, but only seven male patients signed informed consent. Interestingly, about 85% of HIV-infected individuals in Mexico are male; for reasons that are unrelated to HIV status (e.g., child rearing), about 95% of HIV-positive participants in clinical trials are male (29). Due to our limited sample size, we were not able to identify factors associated with response to PDG. Finally, we did not assess mechanism of response. All those questions will be addressed in an ongoing placebo-controlled trial.

In conclusion, our data suggest that add-on therapy with the ACh inhibitor PDG may improve the number of circulating CD4^+^ T-cells in HIV-infected, immunological non-responder patients.

## Ethics Statement

This study was carried out in accordance with the recommendations of the institutional Ethics in Human Research Committee with written informed consent from all subjects. All subjects gave written informed consent in accordance with the Declaration of Helsinki. The protocol was approved by the Ethics in Human Research Committee.

## Author Contributions

SIVF, JCC, and JSM designed the study, analyzed and interpreted data. SIVF, PFBZ, and JCC wrote the manuscript. PFBZ analyzed raw data and performed statistical analysis. CARO, BCD, JAV, and CCB helped collect and interpret data. All authors approved the final version of the manuscript.

## Conflict of Interest Statement

The authors declare that the research was conducted in the absence of any commercial or financial relationships that could be construed as a potential conflict of interest. The reviewer DM and handling editor declared their shared affiliation.
